# Jejunal Intussusception Secondary to a Large Inflammatory Fibroid Polyp: A Case Report and Discussion of Differential Diagnosis

**DOI:** 10.1155/2023/9417141

**Published:** 2023-04-13

**Authors:** Asma Khalid Abu-Salah, Eric Brocken, Hector Mesa, Katrina Collins

**Affiliations:** Department of Pathology, Indiana University School of Medicine, Indianapolis, IN 46202, USA

## Abstract

Inflammatory fibroid polyp (IFP), initially considered a reactive process, is now recognized as a benign mesenchymal neoplasm of the gastrointestinal tract. We report a case of a 68-year-old woman with medically refractory Crohn disease that presented with intussusception requiring surgical intervention. The resection revealed a jejunal mass consisting of a submucosal proliferation of bland spindle cells in a fibrous stroma infiltrated by numerous eosinophils. By immunohistochemistry, the lesion was positive for vimentin and negative for desmin, smooth muscle actin (SMA), S-100, CD117, DOG1, ALK (D5F3), Melan-A, HMB-45, CD34, and STAT6. Ki-67 proliferative index was low (<1%). The mass was classified as IFP by its characteristic morphology and associated eosinophilia. IFP should be considered in the differential diagnosis of adults with intussusception or bowel obstruction. Definitive treatment typically requires surgical resection of the involved bowel segment.

## 1. Introduction

Inflammatory fibroid polyp (IFP) is a rare benign mesenchymal neoplasm that can be found throughout the gastrointestinal (GI) tract but is more frequent in the gastric antrum, followed by the small intestine, in particular the ileum, and is rare at other locations [1–4]. Most intestinal IFPs are asymptomatic, but large IFPs may cause pain, obstruction, and bleeding or act as a lead point for intussusception, especially in children. IFP has been reported in the literature under a variety of names, including polypoid fibroma, submucosal granuloma with eosinophils, and eosinophilic granuloma. In 1953, Helwig and Ranier coined the term inflammatory fibroid polyp [5]. For a long time, it was considered to be a reactive process secondary to infection, inflammation, irritants, or foreign body [6], until recent studies demonstrated consistent mutations in the platelet-derived growth factor receptor alpha (*PDGFRA*) gene, similar to a subset of gastrointestinal stromal tumors and inflammatory myofibroblastic tumors. It is now recognized as a benign mesenchymal neoplasm indigenous to the GI tract [7–12].

## 2. Case Report

**Figure 1:**
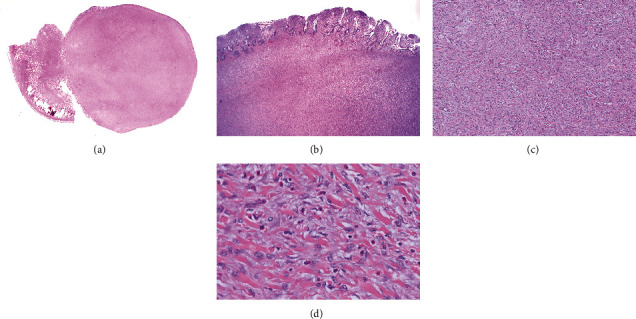
Inflammatory fibroid polyp. (a, b) Low-power magnification showing a predominantly submucosal nodular proliferation with some extension into mucosa comprised of bland spindle-shaped mesenchymal cells, well-vascularized with concentric distribution of inflammatory infiltrate consisting mainly of eosinophils arranged around vessels. (c, d) High-power magnification showing bland spindle cells, small vessels, and eosinophil-rich mixed inflammatory infiltrate. (Hematoxylin and eosin stain: (a) original magnification ×2; (b) original magnification ×10; (c) original magnification ×20; and (d) original magnification ×60).

**Figure 2:**
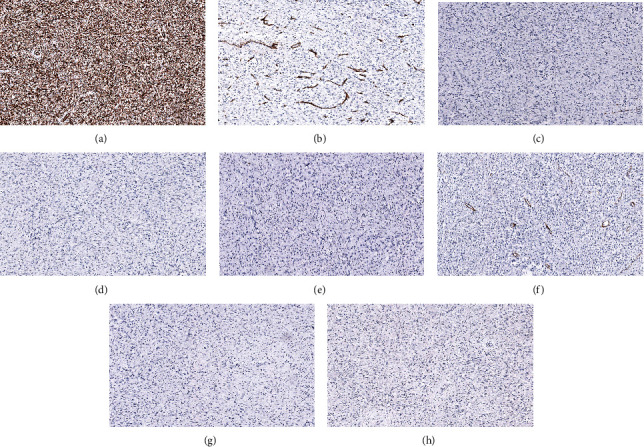
Immunohistochemical staining showed that the spindle-shaped mesenchymal cells were positive for vimentin (a) and negative for CD34 (b), CD117 (c), DOG1 (d), S-100 (e), SMA (f), STAT6 (g), and ALK (D5F3) (h) (original magnification: ×20).

A 68-year-old woman with history of recurrent small bowel obstruction secondary to medically refractory Crohn disease presented with abdominal pain, nausea, and vomiting. Computed tomography (CT) of the abdomen and pelvis demonstrated diffuse wall thickening involving the mid small bowel through the terminal ileum with abnormal dilatation including a 23 cm long segment of distal ileum and mesenteric stranding located about 8-10 cm proximal to the ileocecal junction. A developing fluid pocket and surrounding phlegmon approximately 7 mm in size was noted, suggestive of possible abscess or early fistulous tract formation. The decision was made for ileocolic resection, which showed evidence of a bowel obstruction with dilated proximal small bowel and an unanticipated intussusception in the mid jejunum with a palpable mass. Additionally, there was active ileal Crohn disease involving the last 20 cm of the terminal ileum with associated stricture. Gross examination revealed a 4.2 cm well-circumscribed, gray-white, lobulated mass with a fleshy cut surface in the jejunum. Histologic sections showed a submucosal lesion with focal extension into the mucosa consisting of bland plump spindle cells embedded in a loose fibrous stroma and infiltrated by numerous eosinophils (Figure 1). Immunohistochemical (IHC) studies (Figure 2) showed that the spindle cells were positive for vimentin and negative for desmin, smooth muscle actin (SMA), S-100, CD117, DOG1, ALK (D5F3), Melan-A, HMB-45, CD34, and STAT6 indicative of fibroblastic lineage. Ki-67 proliferative index was low (<1%). The clinical and pathologic findings led to a diagnosis of IFP. Imaging studies done four months postresection showed no evidence of recurrence; however, the patient continued to have active Crohn disease despite maintenance therapy with adalimumab.

## 3. Discussion

To our knowledge, there have been 41 cases of IFP reported to occur in the jejunum to date, the majority of which are individual case reports and a few case series, summarized in Table 1 [1, 8, 13–44]. Of the cases previously described, there was a slight female predominance. Our patient presented at an older age than the mean age reported in the literature (68 years vs. 49 years, range 8–77 years). In many of the cases, symptoms were reported for a prolonged period of time, up to 3 months. The majority presented with abdominal pain (22/36, 61%) and nausea and vomiting (9/36, 25%), followed by obstruction (6/36, 17%), anemia/bleeding (4/36, 11%), diarrhea (4/36, 11%), incidentally found at autopsy (5/36, 14%), and only 1 prior case (6%) presented with intussusception. The mass size in our case was slightly smaller than the mean mass size reported in the literature (4.2 cm vs. 4.6 cm, range 0.5–18 cm), and interestingly, all symptomatic cases of IFPs reported in the literature were greater than 3 cm in size. Recurrence was a rare event. Only one case of recurrence of an ileal lesion occurring in a 2-year-old has been documented [45]. Malignant transformation has not been reported.

IFPs resemble other spindle cell tumors of the GI tract presenting as submucosal or mucosal masses: gastrointestinal stromal tumor (GIST), inflammatory myofibroblastic tumor (IMT), hamartomatous polyps, solitary fibrous tumor (SFT), leiomyoma, and nerve sheath tumors [46–48]. Immunohistochemistry allows an easy separation of IFP from tumors with muscle and nerve sheath differentiation but shows overlap with tumors of fibroblastic, myofibroblastic, interstitial cell, and uncertain lineage, especially those typically positive for CD34. Immunohistochemical staining of IFPs is invariably positive for vimentin and usually positive for CD34; however, tumors arising in the small intestine are often negative for this marker. Since CD34 positive and negative tumors show mutations in *PDGFRA*, they are considered to represent variations of the same tumor and not separate entities [12, 49]. A possible explanation for this discrepant phenotype is the recently proposed origin of IFP from telocytes, a special type of interstitial cells involved in neurotransmission. Most telocytes are submucosal and characterized by a CD34(+)/CD117(-)/PDGFRA(+) phenotype; however, CD34(-)/CD117(-)/PDGFRA(+) telocytes occur in the small intestine and likely represent the precursors for the CD34(-) tumors [50]. There is ample evidence supporting that IFPs arise from telocytes: most IFPs harbor *PDGFRA* mutations, IFPs are common in individuals with germline mutations in the *PDGFRA* gene, and most IFPs express PDGFRA by immunohistochemistry [12, 49–51]. Most mutations occur in exons 12 and 18 at mutational “hot spots”; the frequency of exon 12 mutations is higher in intestinal IFP, and exon 18 mutations are more common in gastric IFPs [4, 10, 12, 52, 53].

GISTs typically present as well-circumscribed gastric masses arising from the muscularis propria [54]. In the small intestine, these tumors can cause intussusception similar to IFPs. Grossly, they are indistinguishable from IFP; however, cystic degeneration, hemorrhage, and necrosis are more common in GIST. Histologically, they differ from IFP in that they are not associated with eosinophilia, can show epithelioid morphology, and can be overtly malignant. Immunohistochemically, IFPs are variably positive for CD34, but negative for CD117. By contrast, GISTs commonly stain positive for CD117, DOG1, and CD34. Molecularly, a subset of GISTs show that *PDGFRA* mutations may lack CD117 expression and are more likely to have epithelioid morphology [55]. There is also a small group of GISTs with typical morphology that is negative for CD117 and DOG1 where genetic analysis is necessary to confirm diagnosis [55–59]. Concerning tumor biology, all GISTs have malignant potential; the risk usually correlates with the size of the tumor and the mitotic activity.

IMTs may also present with intussusception, and the most common sites include the liver and biliary tract, spleen, peritoneum, stomach, and colon-rectum while the jejunum-ileum, duodenum, pancreas, and esophagus are less commonly affected sites [60, 61]. IMT morphologically resembles an IFP and is characterized by a proliferation of spindle-shaped myofibroblasts admixed with inflammatory cells; however, in contrast to IFP, the inflammatory cells are predominantly mononuclear: histiocytes, lymphocytes, and plasma cells. A majority of cases shows variable expression of muscle markers: SMA, muscle specific actin (MSA), and desmin [62]. Approximately, 50% of conventional IMTs show anaplastic lymphoma kinase (ALK) gene rearrangements leading to overexpression of ALK by immunohistochemistry [63, 64]. In contrast to IFPs, IMTs may be locally invasive and may recur [65–67] and in rare cases metastasize [64, 68–70]. IMT with epithelioid morphology is particularly aggressive and has been reclassified as epithelioid inflammatory myofibroblastic sarcoma.

SFTs are exceedingly rare in the gastrointestinal tract [71–82]. Histologically, SFTs are usually hypercellular and consist of spindle cells arranged in a storiform pattern, hemangiopericytoma-like vascular pattern, and bands of hyalinized collagen [83]. In contrast to IFP, it is not associated with tissue eosinophilia and may be overtly malignant. By IHC, it is also typically positive for CD34; however, the now widely available STAT6 immunostain allows recognizing this translocation-associated tumor with high sensitivity and specificity [84].

Among CD34 negative tumors, immunoreactivity for SMA and desmin helps to establish a diagnosis of leiomyoma/leiomyosarcoma. Schwannomas and neurofibromas diffusely express S-100 and SOX10, and perineuromas are positive for EMA, Glut-1, and claudin-1. Perivascular epithelioid cell neoplasms (PEComas) are exceedingly rare in the GI tract, consist of a mixture of epithelioid and spindle cells, and can be recognized by the coexpression of melanocytic (HMB-45, Melan-A, and MiTF) and myogenic markers (SMA and desmin). Similar to SFT, these tumors may be overtly malignant.

Inflammatory polyp of Crohn disease may be considered in the differential diagnosis of IFP when encountered at specific segments of the GI tract, most notably the distal pylorus and distal ileum [18]. Hamartomatous polyps can be found in the small intestine and may be a rare cause of intussusception in adults, commonly in the setting of an inherited syndrome, but can also be sporadic. These lesions are characterized by a disorganized arrangement of indigenous tissue elements to the GI tract and may share overlapping features with other lesions of this region. Diagnostic criteria are based on a combination of personal and family history, endoscopic findings, and histologic features of the polyp. IFPs can be distinguished from a hamartomatous polyp by its typical morphology including spindle cell proliferation often forming perivascular cuffs with the presence of a prominent eosinophilic infiltrate and characteristic CD34 immunoreactivity [85]. Eosinophilic infiltration in the GI tract in the context of mass-forming lesions may create diagnostic confusion with IFP. Eosinophilia may be present in parasitic/fungal infections, inflammatory bowel disease, autoimmune disease, hypereosinophilic syndrome, vasculitis, mast cell disease, Langerhans cell histiocytosis, and other hematopoietic malignancies with *PDGFRA* rearrangements [86].

## 4. Conclusion

IFP is a distinct histologic entity that should be distinguished from other mesenchymal tumors of the GI tract, particularly those consisting of spindle-shaped tumor cells. Immunohistochemical studies performed on the surgical specimen should aid in the final diagnosis. Although the majority of IFPs express CD34, negative cases have been described as atypical IFPs. Activating mutations of *PDGFRA* appear to define this lesion molecularly and are responsible for the recruitment of eosinophils. *PDGFRA* mutations have been detected in both CD34 positive and negative cases suggesting they represent variants of the same entity. Immunohistochemical staining and mutation analysis for *PDFGRA* were not available and a limitation of this case study. Surgical excision is curative for symptomatic IFPs with little risk of recurrence.

## Figures and Tables

**Table 1:** Inflammatory fibroid polyps of the jejunum: cases published between 1950 and the present (including current case).

Reference	*N*	Age/sex	Clinical	Surgery	Size (cm)
Polayes and Krieger, 1950 [13]	1	76/M	Obstruction	Yes	7
Kofler, 1952 [14]	4	29/F	Incidental at autopsy	No	“Pea to cherry sized”
		62/M			
		65/F			
		77/F			
Samter et al., 1966 [15]	1	8/F	Vomiting, diarrhea, obstruction, bleeding	Yes	5
Dalton et al., 1977 [16]	2	17/M	Colicky abdominal pain, iron-deficiency anemia	Yes	2.8
		51/F			9
Williams, 1981 [17]	1	52/F	Intussusception	Yes	4
Navas-Palacios et al., 1983 [18]	1	52/F	Abdominal pain	NA	4
Winkler et al., 1986 [19]	1	61/M	Colicky pain, nausea, vomiting, diarrhea	Yes	4
Kim et al., 1994 [1]	1	52/F	Colicky abdominal pain	NA	NA
Ling et al., 1994 [20]	1	56/F	Abdominal pain, vomiting, diarrhea	NA	NA
Oertli et al., 1994 [21]	1	50/M	Obstruction	Yes	NA
Shih, et al., 1997 [22]	1	66/M	Colicky pain, nausea, vomiting, constipation	Yes	5
Muniz-Grijalvo et al., 1997 [23]	1	56/F	Colicky abdominal pain	Yes	6
Kuestermann et al., 1999 [24]	1	34/F	Colicky abdominal pain, nausea, vomiting	Yes	5
Zager et al., 2001 [25]	1	46/F	Abdominal pain	Yes	3
Sah et al., 2002 [26]	1	45/F	Abdominal pain, vomiting, constipation	NA	NA
Topaloglu et al., 2003 [27]	1	56/M	NA	Yes	5
Bays et al., 2004 [28]	1	17/M	Weakness, iron deficiency anemia	Yes	3
Miyata et al., 2004 [29]	1	64/F	Abdominal pain, alternating diarrhea, constipation	Yes	4.5
Spengler et al., 2004 [30]	1	55/NR	Obstruction	Yes	3
Vijayaraghavan et al., 2004 [31]	1	20/F	Severe abdominal colicky pain	Yes	6
Acero et al., 2005 [32]	2	62/M	Obstruction	Yes	3
		67/M	Incidental at autopsy		0.5
Jabar et al., 2005 [33]	1	34/M	Colicky central abdominal pain, vomiting	No	3
El Hajj and Sharara, 2007 [34]	1	52/F	Abdominal pain, nausea, vomiting	Yes	3.5
Cawich et al., 2008 [35]	1	50/M	Colicky abdominal pain, vomiting, obstruction	Yes	3
Szczepanowski et al., 2009 [36]	1	72/F	Colicky abdominal pain	Yes	3
Yakan et al., 2009 [37]	1	28/F	NA	Yes	NA
Singhal et al., 2010 [38]	1	45/M	NA	Yes	1.5
Liu et al., 2013 [8]	2	NA	NA	NA	NA
Kang et al., 2015 [39]	1	51/F	Abdominal pain, palpable mass-like lesion	Yes	4
Talukder et al., 2015 [40]	1	60/F	Colicky abdominal pain, occasional constipation	Yes	4
Wei et al., 2015 [41]	2	NA	NA	NA	NA
Kao and Chen, 2020 [42]	1	47/M	Abdominal pain	Yes	4.5
Karuhanga et al., 2020 [43]	1	48/M	Abdominal pain, vomiting	Yes	18
Sverrisdottir et al., 2020 [44]	1	25/F	Abdominal pain, anemia	Yes	NA
Current case	1	68/F	Abdominal pain, intussusception	Yes	4.2
